# Partial Discharge Detection Using a Spherical Electromagnetic Sensor

**DOI:** 10.3390/s19051014

**Published:** 2019-02-27

**Authors:** Pietro Romano, Antonino Imburgia, Guido Ala

**Affiliations:** L.E.PR.E. H.V. Laboratory, Department of Engineering, University of Palermo, 90128 Palermo, Italy; antonino.imburgia01@unipa.it (A.I.); guido.ala@unipa.it (G.A.)

**Keywords:** partial discharge (PD), antenna sensor, wireless sensor, PD measurements

## Abstract

The presence of a partial discharge phenomenon in an electrical apparatus is a warning signal that could determine the failure of the insulation system, terminating the service of the apparatus and/or the network. In this paper, an innovative partial discharge (PD) measurement instrument based on an antenna sensor is presented and analyzed. Being non-intrusive is one of the most relevant features of the sensor. The frequency response of the antenna sensor and the features to recognize different PD sources and automatically synchronize them with the supply voltage are described and discussed in details. The results show the performance of the instrument can make a fast and correct diagnosis of the health state of insulation systems.

## 1. Introduction

Partial Discharge (PD) measurements are among the main monitoring techniques used in medium and high-voltage electrical grids. In particular, this kind of measurement provides useful information on the operating state assessment of cables, joints, terminations, and other components installed in high voltage systems [1]. Considering the degrading effect of the PDs phenomena [2], if they are detected on time, the breakdown of service of the entire medium/high voltage system can be avoided. For this reason, PD monitoring of electrical apparatuses during normal operating conditions is a great solution to avoid disservices and to increase the reliability of power systems. In the previous years, those measurements were made by means of wired instruments, which, in some cases, imply the shutdown of the related grid generating direct and indirect economic disadvantages. In recent years, instead, with the aim to overcome the above-mentioned PD measurement issues, several antenna sensors operating in wireless mode have been proposed [3,4,5,6]. The developed antenna sensor reported in Reference [3] is used to measure the PD in power transformers, while that proposed in Reference [4] is used in a metal-enclosed switchgear. In References [5,6], instead, UHF, optical, and HFCT PD probes are used to measure PD in a gas insulated switchgear (GIS). Unlike these PD antennas, which are used in very specific contexts due to their design features, the PD sensor described in this work, even if is designed principally to detect PD on cables, is a general-purpose measurement system able to detect PD signals in different application fields [7]. The novel PD detection system, named Pry-Cam^TM^ Portable, has been developed by Prysmian Electronics on the basis of an original patent developed by researchers from Palermo University [8]. The main features of this PD are the use of an ultra-wide band (UWB) antenna with a substantially flat frequency response in the range of PD detection; an AC phase reference system is also part of the sensor. Furthermore, the small size, portability, and the possibility of remote control of the sensor by a tablet, allowing the operator to stay at safe distance from high voltage parts, are other relevant characteristics of the instrument [7,9]. Another important issue in PD measurement is noise rejection and the ability of recognition and separation of different PD phenomena. More details are given in the following paragraphs in which the adoption of the filters appears useful to facilitate recognition of the different PD phenomena acquired simultaneously, as well as the separation of the PD signals from the noise. This latter aspect has been widely studied during the last years, and several techniques have been developed. In References [10,11], the separation of the PD sources has been obtained by means of the power ratio map based on the spectral characteristics of the signal. With the same aim, Robles et al. [12] proposed an independent component analysis (ICA) algorithm, while in Reference [13], the authors analyze the time delay distribution feature of the signal captured by the antennas in order to separate and locate the PD sources in a substation. In the presented PD sensor, different techniques are adopted in which filters based on FFT and pulse shape cross-correlation analysis are used.

However, in this work, a complete description of the Pry-Cam^TM^ sensor is given, starting from the main features of the adopted UWB Antenna. The response of the sensor by considering different distances, directions, and positions between the sensor and the PD source has also been described. After having discussed the calibration of the PD probe, some experimental tests have been carried out with the aim to evaluate the response of the antenna in the detection of different PD phenomena. Then, another test has been made in order to evaluate, in the case of three different PD phenomena simultaneously detected, the ability of the filters for PD source identification and separation. In the final part of the paper, some applications are reported and discussed. 

## 2. Ultra-Wide-Band Antenna Sensor 

The use of antenna sensors for PD measurements provides several advantages compared to the traditional wired PD measurement tools. During the years, different antenna sensors have been proposed [14,15]. In Reference [14], a general approach to the design of the antenna sensor is presented. The authors describe the behavior of different ultrahigh-frequency (UHF) antenna sensors when measuring electromagnetic pulses radiated by PD activity and evaluating frequency responses and sensitivity on the basis of their dimensional and geometrical characteristics. In Reference [15], the electromagnetic wave generated by PDs is detected by a patch-antenna PD sensor. The frequency response of the patch antenna in terms of output impedance, like many other PD measurement transfer impedances *Z*(*f*), is designed selecting the resonance frequency with the modulus of the amplitude response centered on PD frequency in order to solve sensitivity problems and achieve noise rejection. This approach has the effect of separating PD from noises, but at the same time, it modifies the shape of the PD pulses also in terms of amplitude. Furthermore, these antenna sensors do not acquire signals related to the AC voltage phase, which still has to be achieved with another sensor or even with a wired method. 

**Figure 1:**
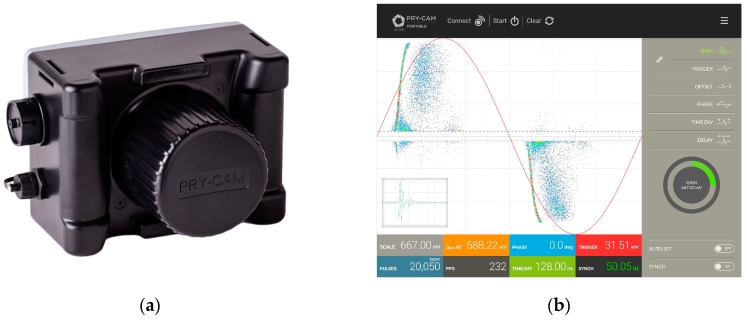
(**a**) Pry-Cam^TM^ Portable and (**b**) a screenshot of the software interface.

The PD measurement system described in this work consists of a portable antenna sensor, also able to receive the AC voltage field signal, combined with a digital acquisition system [16]. The digital system is in turn Wi-Fi connected to a tablet for remote controlling and showing of the acquired signals. The image of the instrument and the graphical interface are shown in Figure 1. 

**Figure 2:**
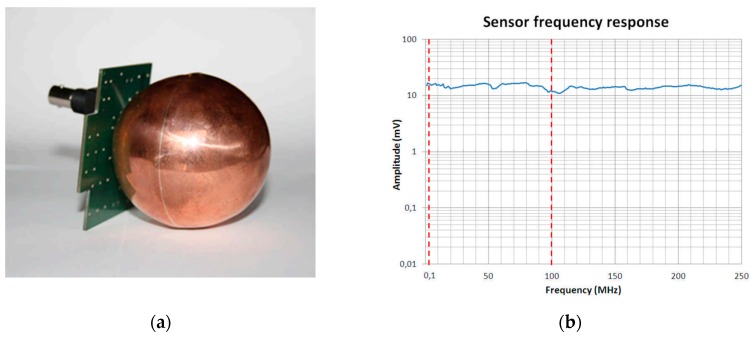
The spherical sensor (**a**) and the frequency response in the range of 0.1 MHz–250 MHz (**b**).

The sensor consists of a conductive sphere over a conductive reference plane, as shown in Figure 2a. The sensor is designed in order to have a 0.1 MHz–100 MHz bandwidth without any relevant peaks in the amplitude profile, so it is non-resonant. In Figure 2b, the frequency response of the sensor in the range of 0.1 MHz–250 MHz, is shown. The sensor frequency response has been obtained in an anechoic chamber using a vectorial network analyzer and by connecting the input terminals with the sensor.

The instrument acquires, stores, processes, and sends all pulse signals to the tablet by a Field Programmable Gate Array (FPGA). The complete hardware specifications, extracted from a technical document of the manufacturer, are reported in Table 1.

**Figure 3:**
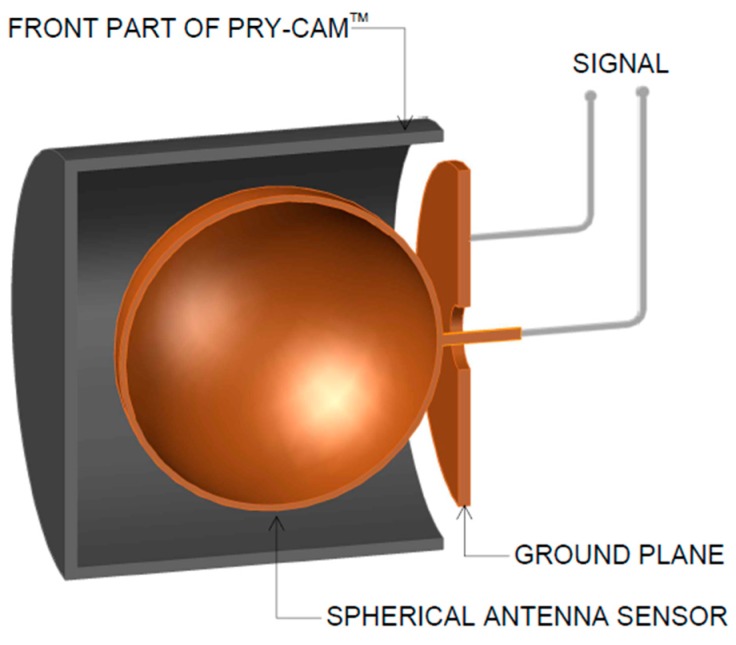
A sectional view of the front side of the Pry-Cam^TM^. (The partial discharge (PD) sensor is subject to patent, and this image is taken from the images contained in Reference [8] as an interpretation of the authors.).

The sensor is inserted in the front part of the instrument, as depicted in Figure 3, and the signal is taken from terminals that are connected between the sphere and the ground plane.

**Figure 4:**
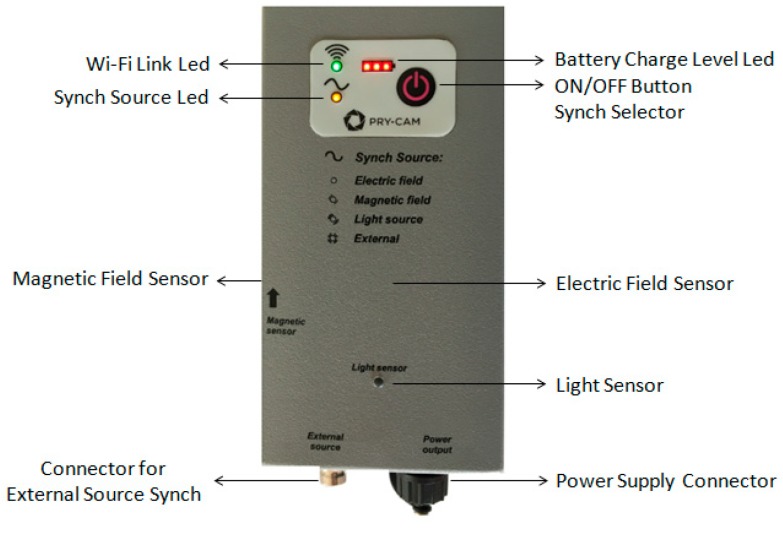
The wireless remote RF synchronizer apparatus.

The total resulting bandwidth of the instrument (sensor + analog front-end + Analog to Digital Converter) is 0.5 MHz–100 MHz, and the absence of relevant peaks enables preservation of the signal characteristics. On the basis of these characteristics, the PD sensor is classified as an ultra-wide band (UWB) system and essentially falls into the category of very high frequency (VHF) electromagnetic PD sensors operating in the near-field region. Moreover, the sensor acquires the AC reference phase information automatically by using the same spherical sensor coupled with an inside low-pass filter. As described in Reference [8]: “The synchronization circuit is an electronic high impedance circuit capable of capacitively detecting, remotely and with no galvanic coupling, the AC voltage supplied to the electronic component being measured, which generates pulse phenomena. This allows synchronization of the detected pulses with the voltage that generates them”. Then, when the PD measures have to be detected, the sensor acquires the electromagnetic field only in the suitable frequency range, without any AC signal superposition. In some cases, such as in the case of shielded components, when the reference voltage is not correctly acquired, the instrument adopts the wireless remote RF synchronizing apparatus shown in Figure 4.

As shown in Figure 4, the remote synchronizer has many sensing features such as magnetic and electric field sensors, a light sensor, and a connector to an arbitrary external source. The Pry-Cam^TM^ Portable can be adopted for PD detection in HV/MV electric components such as cables, joints, terminations, and electrical machines like transformers and rotating machines [7,17]. The behavior of the Pry-Cam^TM^ sensor enables the user to successfully detect the waveform of a typical PD repeated transient radiated electromagnetic field, preserving the information necessary for the subsequent pattern analysis typically carried out by using conducted measure results, and more recently, by radio frequency analysis. The compactness of the probe and its characteristics made it possible to build a portable, low-cost device for PD detection useful for non-conducted measurements, without any interference with the normal service of the apparatus under testing.

**Figure 5:**
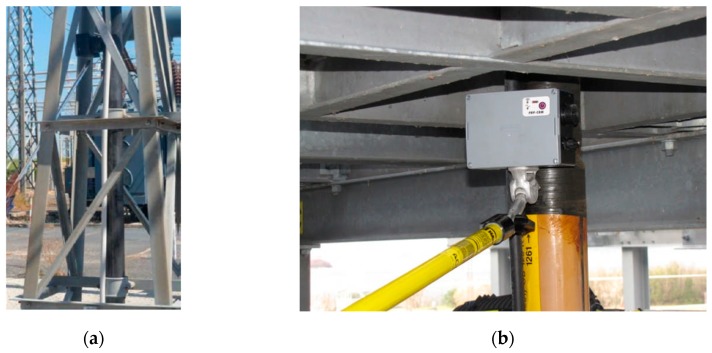
On-site measurements performed by moving the instrument into the proximity of the equipment under testing (EUT) using the hook stick, enabling the operator to be at safe distance. (*These images are extracted from a technical document of Prysmian Electronics s.r.l.*). (**a**) termination earth cable (**b**) cable sheath.

Moreover, the behavior of the antenna sensor frequency response enables the user to precisely detect the various PD activities which may be encountered in an electrical apparatus (corona, surface, etc.), without significant artifacts. Especially in on-field measurements, even if some wired PD detection systems can be used by retrofitting the apparatus with minimum invasiveness, it is necessary to plan some dedicated procedures to be done by expert operators in order to make a safe measuring condition. For example, the insertion of a high-frequency current transformer (HFCT) needs the operator to be in the close proximity to the earth conductor in a fixed safe position; moreover, the AC reference signal needs a further wired connection with the equipment under testing (EUT). On the contrary, the total absence of wired connections characterizing the Pry-Cam sensor improves portability and ease of use without significant operator specialization for its use. In fact, the sensor can be moved in close proximity of the EUT with a simple hook stick so as to keep the operator at a safe distance, as shown in Figure 5.

Furthermore, because the electromagnetic sensor is free of any galvanic connection with the power component where the PD defect sources are placed, the only coupling is given by its distance and orientation. These dependencies will be described in detail in the following paragraphs. No recommendations are given in IEC 60270 [18] for UWB measuring methods, asserting that: “these methods or instruments, in general, do not directly quantify the apparent charge of PD current pulses”. In the next paragraph, the authors describe some considerations on the calibration process which cannot be made in the traditional way due to the wireless connection between the antenna sensor and the PD source.

## 3. Sensor Behavior and Free-Space Radiometric PD Measurements 

It is a fact that in some cases, conventional detection techniques, with the measuring instrument wired-connected to the device under test, are not always capable of accurately detecting and tracking the position of the source, even if PDs are revealed. This problem is particularly evident in cable measurements or in systems where signal attenuation is the major problem. In these cases, the UWB antenna sensor can be very useful. As highlighted by some researchers, there are still no normative references that allow the adoption of these tools in official tests [19,20]. 

In this paragraph, the electromagnetic sensor behavior, dependent on the distance and position between the sensor and emitting source, has been investigated. Even if in practical use, the sensor, especially for PD detection on cables, showed the best result when placed in the close proximity to the cable in order to have a capacitive coupling. Other configurations have also been used due to the specific frequency response of the sensor. Indeed, due to the large flat bandwidth, it is possible to detect, with the same degree of fidelity, the PD signal with a large frequency spectrum ranging from lower to higher frequencies. So, both a radiated electromagnetic field without (near field) and with propagation phenomena can be efficiently detected. In other words, it is not necessary to establish a difference in terms of the capacitive coupling or radiative effects when dealing with the sensor performance, because these phenomena are automatically considered in the intrinsic behavior of the sensor itself. In fact, as shown in the next section, the sensor detects effectively all the various relevant PD activities, whatever the frequency content of the time-domain signal. With the aim of evaluating this behavior, various experimental tests have been done with the standard IEC 60270 basic partial discharge test circuit and with a resistive (50 Ohm) coupling device series connected with a coupling capacitor. 

**Figure 6:**
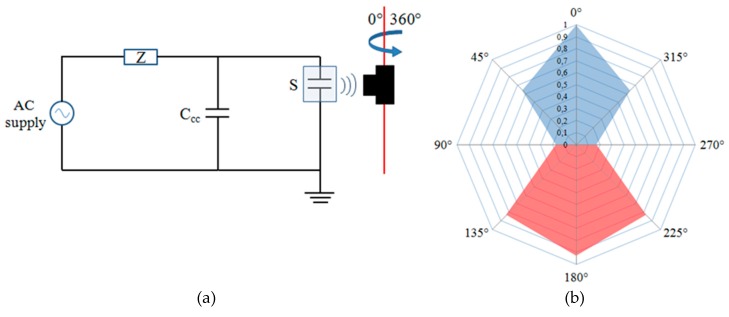
The IEC 60270 test circuit with (**a**) the corona emulator and (**b**) the PD pulse amplitude antenna response.

At first, a sample emulating corona discharges has been used, with classic configuration needle-plane and 5–10 mm air gap, and the sensor has been positioned at 15 cm from the PD source. In order to evaluate the antenna response, according to the rotation angle with respect to the source, the PD pulses have been acquired by rotating the Pry-Cam^TM^ horizontally from 0°–360°, as shown in Figure 6a. The acquired PD amplitudes have been scaled with respect the maximum amplitude of the 0° phase angle, and the amplitude response diagram is reported in Figure 6b.

The red trace on the amplitude diagram represents the polarity inversion of discharges for angles up to ±180°. This behavior means that the sensor is not particularly sensitive to the testing environment. Moreover, the instrument has high sensitivity both on the front and on the back (as shown in Figure 6b). This enables us to better acquire the PD signal from the front and to in turn get the environmental noise from the back. Since the output of the sensor is acquired in a differential way, the result is that the noise taken from the front and the backside will be canceled, thus increasing the SNR of the measure. The small amplitude of the residual noise can be eliminated by adjusting the trigger level. 

**Figure 7:**
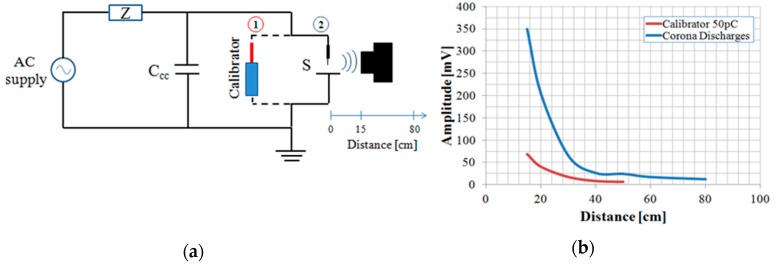
Measurements with the corona emulator and calibrator at different distances from the discharge source: (**a**) Measurement circuit; (**b**) results.

Secondly, some free-space radiometric (FSR) measurements have been made moving the sensor at different distances from the pulse source. Similar measurements have been done by A. A. Jaber et al. in [20]. In this paper, the author says that: “the application of FSR methods to measure the absolute PD intensity (i.e. apparent charge) has been considered to be difficult, if not impossible while possible objectives are to compare the frequency spectrum of radiated PD signals with the spectrum measured by using the electrical galvanic contact method and to establish the plausibility of estimating effective radiated power (EPR) as an alternative measure of absolute PD intensity to apparent charge”. In the present work, only the attenuation coefficient with distance was detected in order to evaluate the antenna performance in free space. The measurements were made with the corona discharge emulator. Then, they were repeated using a calibrator equipped with an antenna, placed in the same position as the corona discharge emulator. The results of both measurements are shown in Figure 7, where the blue line represents the amplitudes of corona discharges detected for distances between 15 cm and 80 cm, while the red line represents the amplitudes detected by the calibrator, set at 50 pC, up to a distance of 50 cm. Beyond these distances, the signal in both cases was no longer detectable.

**Figure 8:**
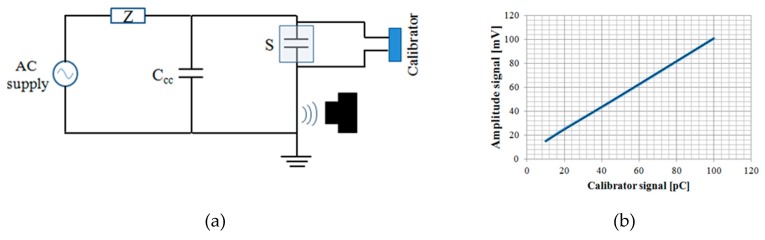
Measurements with the calibrator at different amplitudes of the signal: (**a**) The measurement circuit; (**b**) results.

The last measurements were made with the sensor placed in contact with the earth cable at about one meter from the discharge source. The calibrator was inserted in parallel with the test object (S). This configuration is identical to that of the calibration process for wired systems reported in the IEC 60270 standard; the only difference is that the sensor is placed in contact with the earth conductor, and not with the coupling device (CD). Results are shown in Figure 8, where in Figure 8a the configuration scheme is represented, while in Figure 8b the signal acquired by varying the calibration signal amplitude from 10–100 pC is reported.

The results show a sensitivity of about 1mV/pC, which is in accordance with the manufacturer’s declaration. In the next paragraphs, all measurements have been performed in this last configuration.

## 4. Basic PD Phenomena Characterization: Internal, Surface, and Corona Discharges 

**Figure 9:**
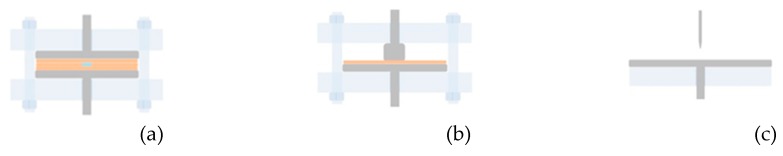
Specimens under testing, simulating (**a**) internal, (**b**) surface, and (**c**) corona defects.

In order to identify different PD phenomena with the Pry-Cam^TM^ sensor, the PD pattern and some characteristics of PD pulses have been acquired and analyzed. The basic phenomena are internal, surface, and corona discharges. To simulate internal discharges, a specimen composed of three overlapped Kapton layers (127mm thickness each) with an air void defect obtained by making a hole in the middle foil has been realized as shown in Figure 9a. The specimen simulating surface discharges instead has been realized by means of a single XLPE layer interposed between two electrodes with different diameters (10 mm and 60 mm), as depicted in Figure 9b. In Figure 9c, the needle-plane specimen (5–10 mm air gap) used to obtain corona discharges is reported.

**Figure 10:**
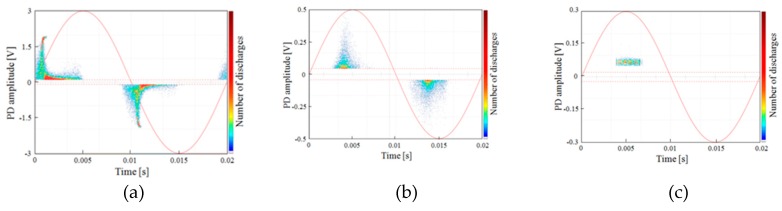
PRPD patterns of (**a**) internal 2.3 kV, (**b**) surface 2.0 kV, and (**c**) corona discharges 3.0 kV.

By using the measurement setup of Figure 8a, the specimens have been stressed with a voltage in the range 2.0–3 kV. The obtained PD patterns are depicted in Figure 9, in which it is possible to distinguish internal (Figure 10a), surface (Figure 10b), and corona (Figure 10c) PDs phenomena. 

**Figure 11:**
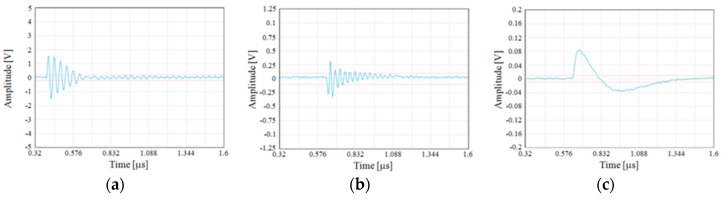
Single PD pulses of (**a**) internal, (**b**) surface, and (**c**) corona discharges.

**Figure 12:**
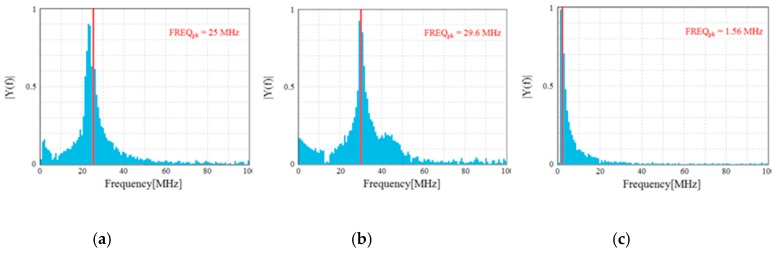
Pulse frequency spectra of (**a**) internal, (**b**) surface, and (**c**) corona discharges.

The detected single PD pulses and the related frequency spectrum, useful to help better identify and recognize the different PD phenomena, are reported in Figure 11 and Figure 12, respectively. In detail, Figure 11a,b show the PD pulses of the internal and surface discharges, respectively. The pulse of Figure 11c is that of the corona PD. The latter pulse has a quite different shape compared to those referred to the internal and surface discharges. These differences are also confirmed by the frequency domain analysis. In fact, the frequency spectrum of the corona PD pulse shows a maximum equal to 1.56 MHz, while for the internal and surface PD pulses, the maximums are concentrated at around 25 MHz and 29 MHz, respectively. Therefore, the surface PD have higher frequency components than the internal discharge, as in the experimental test carried out by J. M. Martìnez-Tarifa et al. in Reference [21]. With reference to the corona PD, compared to the previous two PD phenomena, in our test, a lower value has been obtained, unlike that obtained in Reference [21]; this is due to the different adopted measurement setup.

In the same measurement conditions, a high-frequency current transformer (HFCT) has been used in order to make a comparison between the PD signals acquired with the Pry-Cam^TM^ and a classic PD detection instrument. The tool used in our test is a commercial HVPV HFCT 140-100 with a 350 kHz–19 MHz bandwidth. The HFCT sensor has been clamped in the grounding conductor, such as that connecting the specimen to the earthing network. Therefore, no substantial changes have been introduced in the measurement set-up.

**Figure 13:**
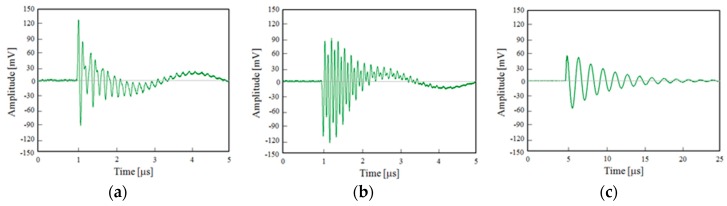
PD pulse of (**a**) internal, (**b**) surface, and (**c**) corona discharges acquired with the HFCT.

**Figure 14:**
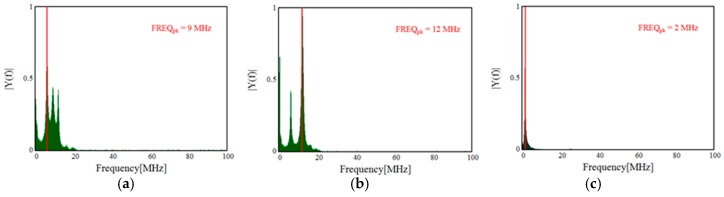
Pulse frequency spectrum of (**a**) internal, (**b**) surface, and (**c**) corona discharges acquired with the HFCT.

Despite this, differences can be seen between the detected PD signals of Figure 13 (provided by the HFCT) and those of Figure 11, due to different features of the HFCT with respect to that of the Pry-Cam^TM^. However, the related frequency spectra reported in Figure 14 show the same trend of those in Figure 12. 

In fact, the surface PD pulse has a higher frequency component in comparison with the other two PD signals (internal and corona), while the corona PD signal has a lower frequency component.

## 5. Simultaneous Presence of Different PD Phenomena

During on-field measurements, the simultaneous presence of different PD sources could occur. Beyond this, many sources of electromagnetic noise are usually present in the surrounding environment, especially in substations, industrial plants, and often also in high voltage laboratories. In this condition, the recognition and separation of PD sources is an ambitious challenge, especially in the case of overlapping patterns.

**Figure 15:**
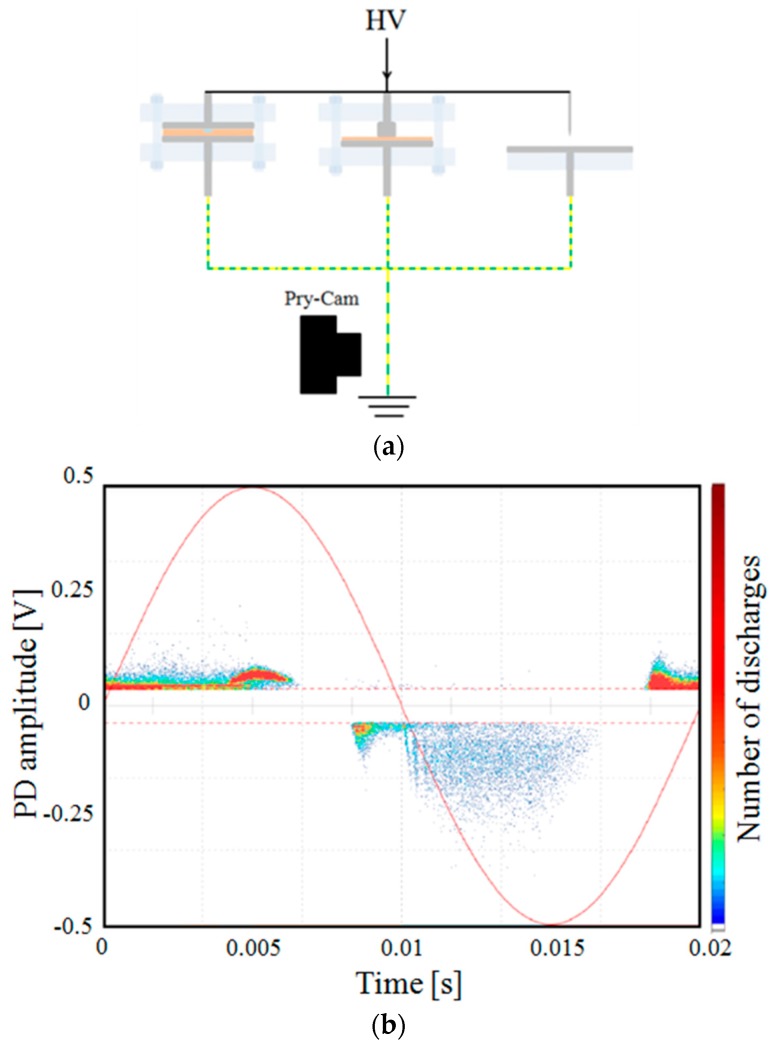
The simultaneous measurement of different PD signals. (**a**) The acquired PRPD pattern of three PD phenomena at 3.0 kV; (**b**) the three specimens under testing are parallel connected and the earth connections are joined among them before the PD sensor.

In order to recognize and separate different PD sources, an experimental test has been performed, and results are reported. In particular, the three specimens of Figure 9 have been parallel connected and powered at a high voltage. The three specimens’ connection is reported in Figure 15a, and the PD pattern provided by the sensor in this configuration is reported in Figure 15b. 

Patterns of different PD sources are totally or partially overlapped, as in the case of internal and corona with surface discharges for the positive half cycle of the applied voltage, and the internal and surface discharges for the negative half cycle.

In order to separate the different PD phenomena from each other, as well as the PD pulses from the noise, the acquisition system of the Pry-Cam^TM^ utilizes particular software filters. Since the various PD activities have different frequency content and time domain profiles, in order to separate the PD phenomena from each other, as well as the PD pulses from the noise, the acquisition system of the Pry-Cam uses filters based on pulse FFT and pulse shape cross-correlation analysis in a time domain. The cross-correlation analysis is applied among one pulse, arbitrarily selected by the operator from the pattern, and all other acquired PD pulses. For this reason, the concept of filtering has to be “read” really as a separation procedure.

**Figure 16:**
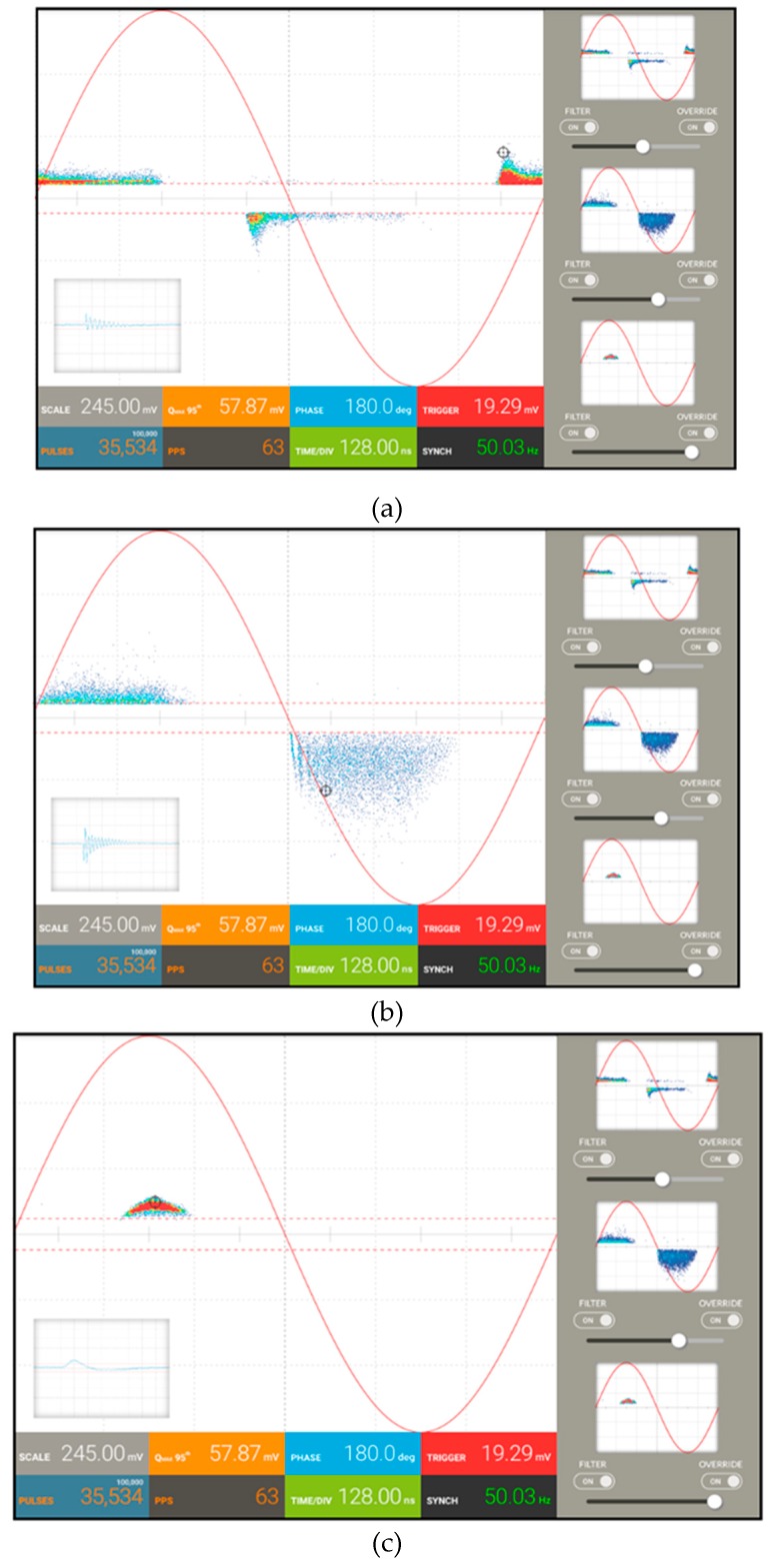
Filter applications on the acquired PD pattern in order to distinguish (**a**) internal, (**b**) surface, and (**c**) corona discharges.

In our experimental test, considering the low magnitude of the noise signals, the setting of the trigger level was sufficient enough to remove it from the PD signals. The separation among the PD sources, instead, has been carried out by using the filters, as reported in Figure 16, in which the screenshots of the acquisition software are depicted.

In order to choose different PD phenomena, filters are applied by selecting a single PD pulse from the pattern with the index finger and then moving the selected pulse to the right side of the screen, where up to three filters can be applied simultaneously. For each of them, there are three cursors that the operator can choose; the filtering intensity by varying the cross-correlation level, the pattern visualization between the selected signal and remaining signals, and finally, the filter off switch. The applied filters can work in real time or after measuring (post-processing mode). In Figure 16a, the surface and corona pulses have been filtered, and therefore only the internal discharges are visible in the PD pattern. In Figure 16b,c, only the surface and corona discharges are present, respectively, while the other PD pulses have been filtered.

## 6. Other Applications of the Antenna Sensor 

As previously reported, the antenna sensor is mainly used to measure the PDs phenomena on cables, but the advantages provided by the sensor have allowed for a variety of different applications. In Reference [7], the PDs measures have been made on an asynchronous three-phase motor and on a synchronous generator machine. While, in Reference [9], the PDs monitoring with the Pry-Cam has been performed on a 400 kV XLPE cable joint. Beyond the above reported industrial applications, some other research activities, developed by the authors, are in summary described in the following paragraphs.

### 6.1. The Detection of PD Under Square Wave Voltage 

PD measurements under square wave voltage with high gradient flanks, typically provided by power electronic devices, required specific instruments and particular measurement set-ups to be performed. In Reference [22], the authors carried out the tests by means of both the traditional stochastic PD detection system and a prototype of the Pry-Cam^TM^ called the Portable Antenna Sensor (PASPD). The obtained results show that the acquired PRPD patterns with both PD measurements systems are in good accordance, and even if the synchronization process with PASPD is not easy for higher frequencies, both instruments can distinguish PD from noises.

**Figure 17:**
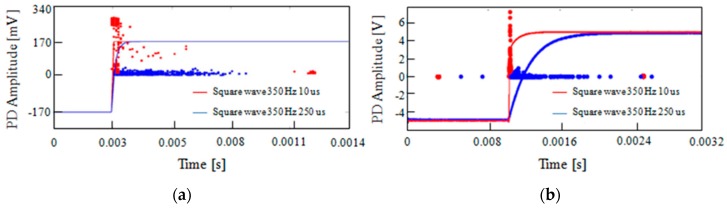
The positive discharge obtained with (**a**) PASPD and (**b**) STOPD systems obtained with square waves of 350 Hz and 10 μs and 250 μs rise times.

For example, in Figure 16, the PD patterns provided by the Pry-Cam^TM^ and by the stochastic PD detection system (STOPD) are reported in Figure 17a,b respectively. In this case, the specimen under testing was a twisted pair in which two square wave voltages of 350 Hz and different rise times of 10 μs (red pattern) and 250 μs (blue pattern) were applied separately. As can be seen, for both PD detection systems, a similar trend of the PD patterns was obtained. 

### 6.2. The Continuous Periodic DC Waveform

**Figure 18:**
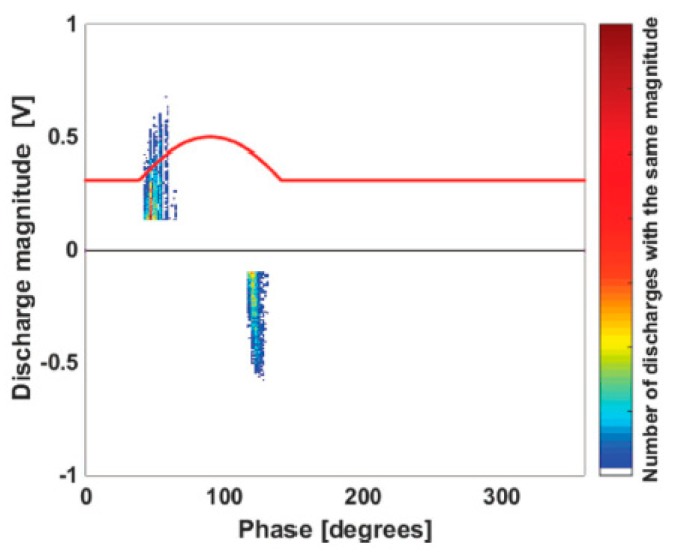
The internal PD pattern acquired using the continuous periodic DC waveform.

The antenna sensor was also used to detect PDs signals in an experimental test in which a specimen was stressed with a continuous periodic DC waveform [23]. This new type of waveform has been developed with the aim to overcome some PD measurements issues under DC stress. It has been derived from a single-phase half-wave rectifier and follows a sinusoidal law for a certain period while it is constant in the remainder part. The sinusoidal part of the waveform allowed both the triggering of the PDs and the PD probe synchronization with 50 Hz. As part of the advantages provided by the proposed new waveform for the PD measurement, it is important to highlight that the antenna sensor results are able to detect the PD pulses and provide a satisfactory PD pattern. For a specimen containing an air void defect, the acquired PD pattern is reported in Figure 18, in which the red line represents the continuous periodic DC waveform used as the voltage stress.

### 6.3. Simultaneous Measurement of PD and Space Charge 

Under DC stress, the PDs phenomena are strongly influenced by the accumulated space charges in the surface of the defect, and vice-versa. For this reason, in Reference [24], simultaneous measurements of PDs and space charges were made. To achieve this, a specimen containing an air void defect was inserted inside the pulsed electroacoustic (PEA) cell, the instrument used to measure the space charge [25]. In order to measure the PDs, the conventional methods, in which the measurement impedance Z is connected in series to the sample, cannot be adopted. This is because the electrical PEA circuit is characterized by the fact that all components must have the same reference to the ground, and thus, the Z results are short-circuited by the various ground connections. Therefore, there were other preferential ways for the reclosure to ground the discharge pulses, and thus the measurement impedance was not affected by any electrical signals. The solution was to measure the discharge pulses directly from the source in a radiated way by means of the antenna sensor. 

**Figure 19:**
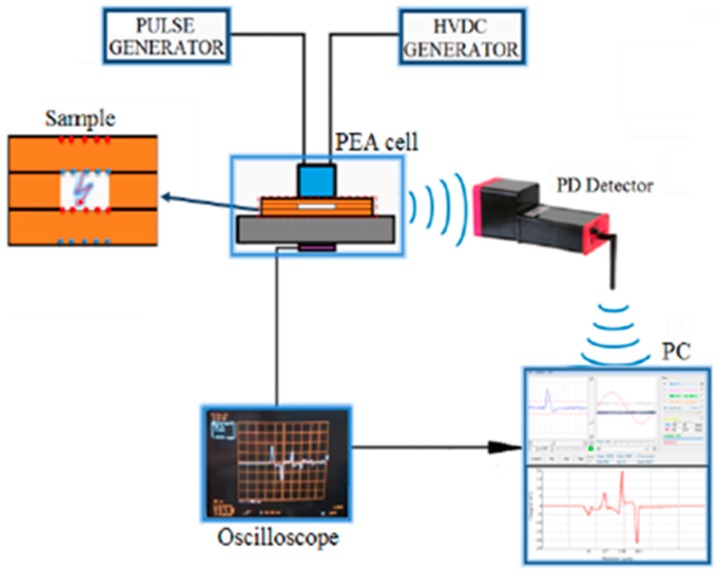
The measurement setup for the simultaneous detection of the space charge and partial discharges.

The adopted measurement setup is reported in Figure 19. The HVDC generator is used to stress the sample placed within the PEA cell, while the pulse generator is used to vibrate the charges deposited into the sample interfaces. The charge vibration generates pressure waves, which reach a piezoelectric sensor placed in contact with the ground electrode of the PEA cell. The PEA cell output signal, which is the signal coming from the piezoelectric sensor, is proportional to the charges, and it is displayed by the oscilloscope. Simultaneously, and for voltage stress greater than the PD inception voltage, the PD signals are detected by a prototype of the Pry-Cam^TM^. 

**Figure 20:**
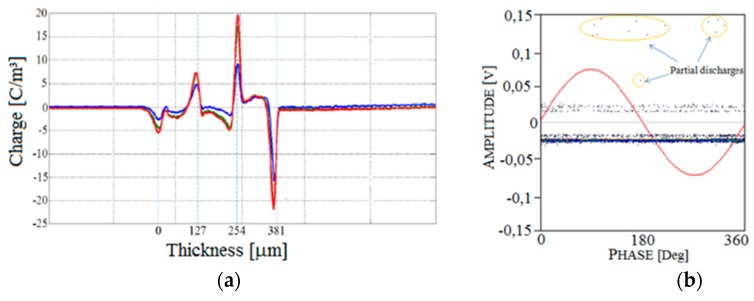
Simultaneous pattern acquisition of (**a**) the space charge and (**b**) the partial discharges. The red line is the AC reference voltage that is displayed permanently.

Finally, both space charges profiles (Figure 20a) and PDs signals (Figure 20b) are sent to a computer in which they are represented on the same plane simultaneously.

## 7. Conclusions

In this work, a complete description of the recently developed Pry-Cam^TM^ sensor has been reported. Starting from the main features of the adopted antenna, the behavior of the sensor based on the different distance, direction, and position between the sensor and the PD source has been considered and described. Experimental tests show that the better-detected PD signal, in terms of magnitude, is obtained when the PD probe is placed in front of the PD source end at a distance of about 15 cm. On the contrary, a weak signal is sensed when the angle between the PD probe and the PD source is equal to 90 and 270 degrees. Further measurement results show that the antenna sensor is able to detect internal, surface, and corona discharges. The obtained PD patterns are in good agreement with those typically provided by the traditional wired PD measurement systems. In addition, with reference to the separation of the different PD phenomena simultaneously detected, the performance of the filters based on pulse FFT and pulse shape cross-correlation analysis have also been validated. In conclusion, although the new antenna sensor has been designed to measure PD in cables, its features also allow it to monitor the PD in different fields of applications in both radiated and in-contact ways. 

## Figures and Tables

**Table 1:** Hardware specifications ^1^ of the Pry-Cam^TM^.

**Sensor**	
Type:	Electromagnetic, based on a patented Ultra Wide Band antenna, also providing AC synch signal
Bandwidth:	0.5–100 MHz
PD sensitivity:	Down to 1 pC
Synch sensitivity:	Down to about 150 VAC (at 10 cm)
Synch frequency:	From 10 Hz to 1 KHz
**Acquisition Unit**	
Sampling frequency:	200 MS/s
Bandwidth:	100 MHz
Gain:	From 0 dB to 40 dB
Trigger:	Digital, fully configurable
Synch resolution:	16 bit (5 μs)
Timestamp resolution:	5 ns
Processing:	Real-time filtering, high-speed pattern only, TDR
**Repetition Rate**	
Full pulse waveform:	Ethernet >10,000 pps, WiFi: >3,000-6,000 pps
Pattern only:	Ethernet >50,000 pps, WiFi: >10,000 pps
Interfaces:	Wireless 802.11 b/g (WiFi), Optical Fiber Ethernet (100-Base FX, optional)
Remote Synch:	Wireless RF interface @ 868 MHz
Working mode:	Local, remote and monitoring
Power supply:	12 V, 200 mA
Backup battery:	Li-Po 7.4 V, 2200 mAh
Autonomy in battery mode:	About 5 h
Weight:	About 400 g (depending on options)
Working temperature:	From −25 °C to 70 °C
Dimensions:	160 mm × 120 mm × 130 mm (LxWxH)
Case:	Rugged ABS plastic with IP67 protection rating

*^1^ this data are extracted from a technical document of Prysmian Electronics s.r.l..*
